# Nordic walking training in elderly, a randomized clinical trial. Part II: Biomechanical and metabolic adaptations

**DOI:** 10.1186/s40798-019-0228-6

**Published:** 2020-01-13

**Authors:** Natalia Andrea Gomeñuka, Henrique Bianchi Oliveira, Edson Soares da Silva, Elren Passos-Monteiro, Rodrigo Gomes da Rosa, Alberito Rodrigo Carvalho, Rochelle Rocha Costa, Martín Cruz Rodríguez Paz, Barbara Pellegrini, Leonardo Alexandre Peyré-Tartaruga

**Affiliations:** 1 Exercise Research Laboratory, Universidade Federal do Rio Grande do Sul, 750 Felizardo Street, Porto Alegre, 90690-200 Brazil; 2 Departamento de Investigación de la Facultad de Ciencias de la Salud, (UCAMI) Universidad Católica de las Misiones, Posadas, Argentina; 3 Post-Graduate Program in Health Sciences, Federal University of Health Sciences, Porto Alegre, Brazil; 4 Physical Therapy College, Universidade Estadual do Oeste do Paraná, Cascavel, Brazil; 5 Polytechnic Institute San Arnoldo Janssen, Posadas, Argentina; 6 CeRiSM (Research Centre of Mountain Sport and Health), University of Verona, Rovereto, Italy

**Keywords:** Walking with poles, Inverted pendulum recovery, Cost of transport, Aging, Oxygen consumption, EMG co-activation, Mechanical energy fluctuations

## Abstract

**Background:**

Nordic walking is an attractive method of endurance training. Nevertheless, the biomechanic response due to the additional contribution of using poles in relation to free walking training has been less explored in the elderly. Purpose: This randomized parallel controlled trial aimed to assess the effects of 8 weeks of Nordic walking and free walking training on the walking economy, mechanical work, metabolically optimal speed, and electromyographic activation in elderly.

**Methods:**

Thirty-three sedentary elderly were randomized into Nordic walking (*n* = 16) and free walking group (*n* = 17) with equalized loads. Submaximal walking tests were performed from 1 to 5 km h^−1^ on the treadmill.

**Results:**

Walking economy was improved in both free and Nordic walking groups (*x*^2^ 4.91, *p* = 0.014) and the metabolically optimal speed was increased by approximately 0.5 km h^−1^ changing the speed-cost profile. The electromyographic activation in lower and upper limbs, pendular recovery, and total, external, and internal mechanical work remained unchanged (*p* > 0.05). Interestingly, the internal mechanical work associated with arm movement was higher in the Nordic walking group than in the free walking group after training, while the co-contraction from upper limb muscles was reduced similarly to both groups.

**Conclusions:**

Eight weeks of Nordic walking training effectively improved the walking economy and functionality as well as maintained the gait mechanics, similar to free walking training in elderly people. This enhancement in the metabolic economy may have been mediated by a reduction in the co-contraction from upper limb muscles.

**Trial registration:**

ClinicalTrails.gov NCT03096964

## Key Points

- Individualized and periodized walking training using poles improves the walking economy without modifying the mechanical work of walking in elderly.
- The metabolically optimal speed (speed where the metabolic cost is lower) is increased from these training programs.
- The improved walking economy is associated with a reduction of co-contraction from upper limb muscles.

## Introduction

It is well established that the increased motor variability in elderly is associated with risk of falls and low functional mobility [1], and the lack of physical fitness is a strong predictor of mortality [2, 3]. It has been determined that the slower walking speed of the elderly is associated with the muscle weakness and in turn, the reduction on a range of joint motion and biomechanic changes resulting in a safer walking strategy [4]. Many changes in locomotion pattern and neuromuscular control occur in old age, altering segment trajectories, thus resulting in greater augment of internal mechanical work (*W*_int_), compared with young people [5]. Also, Mian et al*.* [5] showed that muscle co-activation explains the higher metabolic cost of walking (*C*) in elderly in contrast to young people. Therefore, it has been suggested that endurance training can result in benefits through the reduction in the activation of the propeller antagonist muscles of lower limbs [1, 6]. In order to avoid these adverse events and the problems arising from the sedentary lifestyle, there are increasingly more exercise programs for elderly. It is expected that through proper physical activity the elderly improve their mobility and increase their daily life activities [7].

Furthermore, movement patterns that lead to mechanical energy fluctuations of the body center of mass (BCoM) are essential for detecting gait alterations of the elderly. These adaptations from walking interventions should test if an improvement in the metabolic economy of walking [6, 8] is accompanied by enhanced pendular mechanism [9] or due to an increase in self-selected walking speed of the elderly [10–12]. Recently, studies with Nordic walking (NW) training have been carried out in older adults [6, 8, 13–18]. NW is defined as walking with poles, and it is also considered as a physical activity for adults and older people [18, 19]. Some studies evaluated the effect of NW training on physiological and neuromuscular parameters in the elderly [1, 6, 8, 13, 15, 17, 20]; however, the specific adaptations of mechanical work and muscular activation are not fully determined. The upper limb muscles are more intensely activated using poles than without poles [1, 21, 22]. This greater upper limb engagement causes, physiologically, higher oxygen consumption and heart rate [15, 21, 23]. Moreover, the NW causes a larger stride length (even at the same speed) and faster freely chosen speed [8, 9, 22]. Finally, these factors may collectively improve the functional ability of the elderly.

The purpose of the present study was to assess, by means of a randomized clinical trial, the effects of 8 weeks of NW and free walking (FW) training on the following parameters evaluated during walking without poles: *C*; optimal walking speed; heart rate; rating of perceived exertion; *W*_int_; total mechanical work (*W*_tot_), external mechanical work (*W*_ext_); *W*_int_ of trunk, legs, and arms (*W*_int_trunk_, *W*_int_legs_, *W*_int_arms_); pendulum-like recovery (*R*); and surface electromyographic activation (EMG) and co-contraction from three pairs of muscles (deltoideus anterior (DA); triceps brachii (TB); vastus lateralis (VL); biceps femoris (BF); tibialis anterior (TA); and gastrocnemius medialis (GM)) in elderly people. We hypothesized (i) at a group level that NW program would elicit most reductions in the *C* than in FW program due to the higher muscular recruitment of upper limbs using poles and (ii) the *W*_tot_ would remain unchanged and the muscular co-contraction would decrease in both groups consistent with previous observational study [5].

## Materials and Methods

### Study Participants

Thirty-three sedentary elderly people were divided into two groups randomly. The intervention group performed NW training (*n* = 16) and the control group performed FW training (without poles, *n* = 17), both during 8 weeks (see the flowchart of the study in results). The inclusion criteria of the study were sedentary elderly people or non-practitioners of systematized physical activity in the previous six months, aged between 60 and 80 years old, who did not present chronic pain, not even presence of migraine or nausea in daily life, without history of labyrinthitis, non-smoking, and also without factors that could impair the elderly to conclude the training sessions and tests.

Simple randomization of the allocated participants was done using a binary random list (www.randomization.org). A blinded evaluator (an impartial researcher who was not involved in the study, in order to maintain allocation confidentiality and blinding) accomplished allocation concealment through a sequential numbered list, in which each number corresponding to a subject, and thus indicated to which group this subject was included. The randomization process and allocation of the study participants were only carried out after the conclusion of the familiarization with the NW technique and before the beginning of the training period.

All participants read and signed an informed consent form before starting their participation in the study (number 878.736 of ethics committee). All the assessments and the training sessions were performed at the Physical Education, Physiotherapy and Dance School of the Federal University of Rio Grande do Sul (UFRGS) of Porto Alegre, Brazil. This trial has been registered in clinical trials (Number 03096964).

### Trial Design and Procedures

**Fig. 1:**
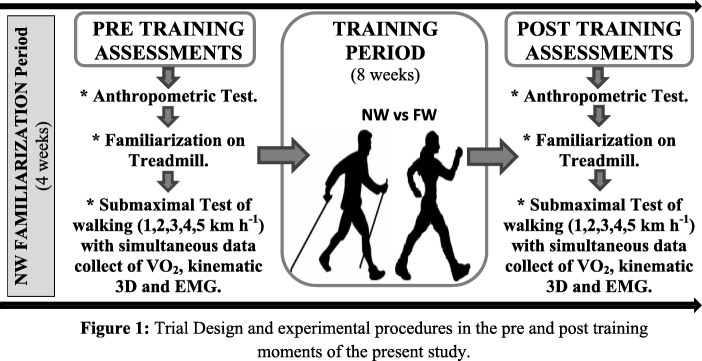
Trial design and experimental procedures in the different moments of the present study

This study was designed as a controlled randomized clinical trial in parallel, with an allocation index of 1:1, according to the CONSORT recommendations [24]. No changes in methods after trial commencement were made (see trial design and procedures in Fig. 1). The present study is the second part of this randomized clinical trial (NCT03096964).

**Fig. 2:**
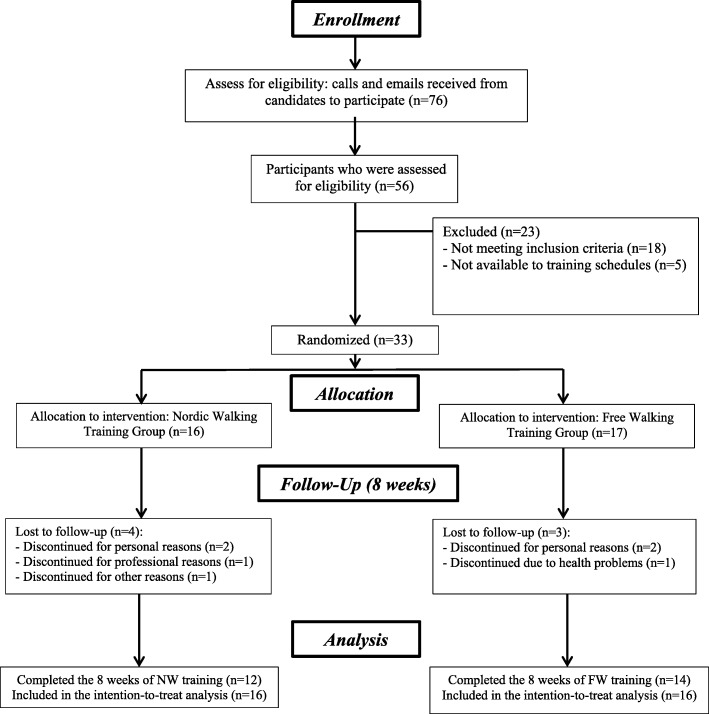
Flowchart of the study participants

The recruitment was carried out after the research had been published in local newspapers, online networks, and posters around the city. After that, 76 people communicated by telephone and were informed of the inclusion and exclusion criteria. Of these, 56 people conducted interviews and of these, 33 were selected to participate in the study and were randomized (see Fig. 2). The recruitment, training, and data collections took place in 2015 (familiarization period (August), pre-training data collection (September), training period (October/November), and pre- and post-training data collection (December)). Initially, all the elderly performed a familiarization period of 4 weeks with the technique of NW (one weekly session of 45 min) and only after that, the participants attended the lab for executing the assessments corresponding to the pre-training period. Recent findings confirm that the correct technique of NW improves the effectiveness of the exercise [25, 26].

On the first day that the participants attended to the laboratory (visit 1), anthropometric assessments, familiarization with the treadmill, and explanation of the procedures of the experimental protocol were performed. In both pre- and post-training, two trained researchers carried out anthropometric assessments. On the second day (visit 2), the elderly performed the incremental walking test on the treadmill. On the third day (visit 3), the participants performed the submaximal walking tests on the treadmill, where we acquired the oxygen consumption, the kinematic data (3D), and EMG data.

After the pre-training assessments, the participants were randomized into the NW group or FW group and executed the training period of 8 weeks. After the training program, the elderly returned to the laboratory to perform the same evaluations of pre-training assessment (post-training). Throughout the intervention period, the participants of the study were instructed to maintain their prescribed medications and their eating habits as usual. The data processing was carried out by trained evaluators who were blinded to the allocation of the participants (see Fig. 2).

### Treadmill and Borg’s Scale Familiarization

In the first visit to the laboratory, the individuals were familiarized with the motorized treadmill (model ATL Inbrasport, Medgraphics, Ann Arbor, USA) and informed about the safety mechanisms, and they walked on the treadmill at different speeds (with gradual increases of 0.5 km h^−1^), thus being familiarized for approximately 15 min. In this visit, the participants were familiarized with Borg’s Scale from 1 to 10 [27, 28], indicating the rating of perceived exertion at each walking speed tested and, also, they were familiarized with neoprene mask that would be used to collect the oxygen consumption. We performed the treadmill speed calibration at different speeds according to the protocol of Padulo et al. [29], and our speed error was less than 0.1%.

### Incremental Test on Treadmill

In the second visit to the laboratory, the participants underwent the incremental maximal test, with the protocol proposed by Bruce et al. [30]. The oxygen consumption, carbon dioxide, and pulmonary volume were measured by indirect calorimetry throughout an automated gas analysis system validated accordingly [31]. The gas analyzer was calibrated before each testing session. The ventilatory data were collected during the test (VO2000, Medgraphics, St. Paul, MN, USA). The test ended when the participants communicated to the researchers through visual signs that they needed to stop the test or if the predicted maximum heart rate was reached or if the respiratory exchange ratio was higher than 1.15. During maximal tests, the volunteers were verbally encouraged to reach their maximal effort.

Subsequently, we determined the values of the maximal oxygen consumption, ventilatory thresholds, and submaximal and maximal heart rates. The heart rate associated with the second ventilatory threshold was used for determining the session intensities in the periodization of training of both groups.

### Measurement During Submaximal Walking Test on Treadmill

In the third visit, the measurements of the EMG were performed with the following sequence: the muscles of interest, DA, TB, VL, BF, TA, and GM muscles were located, and then the trichotomy and asepsis of the skin were performed by abrasion with alcohol and cotton in the region, to decrease the skin impedance. Pre-amplified electrodes with bipolar configuration were longitudinally fixed on the muscles, according to muscle fibers direction and at an inter-electrode distance of 20 mm. A reference electrode was fixed in the clavicle tuberosity. The electrodes were positioned following the recommendations of SENIAM (http://www.seniam.org/). The resistance level between the electrodes and the skin was kept below 3.000 Ohms, which was evaluated by a multimeter (model ET-2652 MINIPA, São Paulo, Brazil). After the first test, paper-film and indelible pen were used to mark the positioning of the electrodes for the location in the subsequent tests [32]. Then, the participants performed the maximal voluntary contractions test of all analyzed muscles and executed three attempts of 10 s, with intervals of 2 min of recovery among each new maximal voluntary contraction repetition.

The EMG data were collected by two electromyographs Miotool 400 (Miotec Equipamentos Biomédicos Ltda, Porto Alegre, Brazil), and the sampling frequency of 2000 Hz for each channel was used. Surface bipolar electrodes of the brand Kendall were used (Meditrace 100; Ag/AgCl; diameter of 10 mm with fixing adhesive). For pin electrodes, the recommendations of SENIAM–BIOMEDII, European Union, were obeyed. After the end of the maximal voluntary contractions recordings, the participants were prepared for the assessment of kinematic data.

For kinematic collection, the participants were prepared with 36 reflexive markers (14 mm, Vicon Biomechanics Marker Accessories) on the anatomic points of interest following the Plug-in-Gait Full-Body model (Nexus 1.8.5 software) of the Vicon Motion Capture 3D kinematic system (Vicon Motion Capture System, Oxford, UK), with three Bonita (1 megapixel) and three T-10 (1.3 megapixel) cameras. The sampling frequency of the cameras was 200 Hz. The dynamic and static calibration was performed to determine the three-dimensional space in which the individuals walked accurately.

The anthropometric measurements used as input data for the model construction were body mass (kg), height (mm), shoulder diameter (mm), elbow diameter (mm), wrist diameter (mm), hand width (mm), knee diameter (mm), and ankle diameter (mm). Once the reflexive markers were positioned, the static collection of the participants was performed on the treadmill, for the subsequent reconstruction of the images. After the reconstruction of the dynamic capture data, the system provides a matrix of 132 columns, three for each reference point (x, y, z) of each marker.

The participants underwent walking tests of 5-min duration at different randomized speeds (1, 2, 3, 4, and 5 km h^-1^) interspersed with 5min of recovery among them. For the simultaneous measurement of the kinematic and EMG data, an additional camera was used, perpendicularly positioned to the treadmill. The camera filmed a light signal (led) that was activated as synchronism (Sinc channel of Miotool) together with the beginning of kinematics and EMG recordings, in the fourth minute of each walking test. The EMG and kinematic data recording took a duration of 30 s.

Before the walking tests, the resting oxygen consumption was collected, in the orthostatic position for 6 min. During the exercise, oxygen consumption and carbon dioxide production were recorded during the whole walking test. Heart rate was measured by cardiac monitors (POLAR, model S 610, Kempele, Finland) and the rating of perceived exertion was registered by using the modified Borg’s scale of 1:10 [28]. The participants walked on the treadmill without poles in pre- and post-training tests in order to test them in conditions closer to daily life.

### Signal Processing

The raw EMG data were exported from Miograph software to compatible files with the SAD32 software (.sad; Data Acquisition System 32 bits, developed by the engineering school—UFRGS, Porto Alegre, Brazil) for subsequent analysis. According to the time obtained by the kinematics of the touchdown and take off moments, a data window corresponding to ten strides was obtained for each test. The determination of the touchdown and takeoff events were based on the kinematics of the foot (error lower than 5%) [33].

Subsequently, the data were analyzed in a mathematical routine created in the LabVIEW® software (National Instruments, Austin, USA 2013). A Butterworth bandpass filter 50–400 Hz, of the 4th order, was applied to rectified data, and a smoothing of the curve with the Hamming’s window technique mathematical model was performed. Mean and standard deviation (mean ± SD) of the EMG normalized by percentage maximal voluntary contractions of pre- and post-training and co-contraction between the muscles DA/TB, VL/BF, and TA/GM were then calculated, and time interpolated by stride cycle. The co-contraction was calculated as the overlap in the area between two muscle pairs, as previously described [34].

The kinematic data corresponding to the same ten steps used in the EMG analysis were processed by routine created in the Matlab® software where the BCoM position and center of segmental masses were calculated, according to Pavol anthropometric tables for elderly people [35]. The kinetic energy (KE = 0.5 M *v*_BCoM_^2^) and gravitational potential energy (PE = Mgh_BCoM_) of BCoM were determined by calculating *v*_BCoM_ (instantaneous speed of BCoM in the sagittal plane with respect to the treadmill), *h*_BCoM_ (height of BCoM in the vertical direction) and by knowing M (the subject’s body mass) and g (gravitational acceleration – 9.81 m s^−2^).

The work necessary to sustain KE and PE changes was estimated by calculating respectively the sum of positive increments of KE and PE. The total external energy of BCoM due to its motion in the sagittal plane was calculated by the instantaneous sum of PE and KE, as previously employed in Gomeñuka et al. [36]. The *W*_ext_ or the work required to raise and accelerate BCoM relative to the environment was calculated as the sum of the positive increments of total external energy [36–38]. The negative mechanical energy changes were not computed here, considering that the cost of negative mechanical work is minimal in comparison to positive work.

1 $$ {\mathrm{W}}_{\mathrm{ext}}=\Delta {E}_{ext} $$

In order to calculate *W*_int_ or the work required to accelerate the segments relative to BCoM, the quantification of KE changes of each segmental center of mass relative to BCoM was used, KE_i_, which is obtained from the sum of its translational and rotational energy, according to Eq. 2:

2 $$ {E}_{\mathrm{int}}(t)=0.5\;{m}_i\;{v}_{ap,r}^2(t)+0.5\;{m}_i\;{v}_{v,r}^2(t)+0.5\;{m}_i\;{w}_i^2\;{k}_i^2 $$

where *m*_*i*_ is the mass of the *i* segment; *v*_*ap,r*_^2^ and *v*_*v,r*_^2^ are the anterior-posterior and vertical velocity of the center of mass of the *i* segment relative to BCoM, respectively; *ω*_*i*_ is the rotational velocity of the *i* segment; and *k*_*i*_ is the radius of gyration of the *i* segment. To calculate *W*_int_, the transfer of energy among thigh and leg segments was assumed, as well as among arm and forearm of the same side of the body, as the Eq. 3.

3 $$ {W}_{\mathrm{int}}=\varDelta {E}_{int} $$

In order to account separately for the contribution of the trunk, arms, and legs, *W*_int_trunk_, *W*_int_arms_, and *W*_int_legs_ were separately calculated by summing the positive increments of the KE curve of the trunk, arms, and legs, respectively, according to Pellegrini et al. [22]. Therefore, the *W*_tot_ is the sum of ǀ*W*_int_ǀ and ǀ*W*_ext_ǀ modules. The mechanical energy exchange between vertical and horizontal energies of the BCoM was also quantified by calculating the percentage *R*, which accounts for how much mechanical energy can be saved through the pendular mechanism of locomotion, according to Eq. 4 [36, 38].

4 $$ R=\frac{W_v+{W}_f-{W}_{ext}}{W_v+{W}_f}\times 100 $$

where *W*_*v*_ is the absolute value of the vertical external work calculated by the variations in PE and vertical KE, *W*_*f*_ is the absolute value of the horizontal *W*_ext_ calculated by the variations of horizontal KE, and *W*_ext_ is the absolute value of external work. The percentage of instantaneous reconversion in the stride cycle was also calculated, *R*(*t*), as proposed by Cavagna et al. [39], as in Eq. 5.

5 $$ R(t)=\frac{\mid {W}_v(t)\mid +\mid {W}_f(t)\mid -\mid {W}_{ext}(t)\mid }{\mid {W}_v(t)\mid +\mid {W}_f(t)\mid}\times 100 $$

The submaximal heart rate and oxygen consumption were averaged from the last 60 s of the test. The walking *C* was indicated in J kg^−1^ m^−1^ described by Saibene and Minetti [40] and also as explained in detail in Gomeñuka et al. [40, 41]. We subtracted the exercise metabolic rate by the stand metabolic rate to find the net metabolic rate and, in turn, we multiplied by speed and transformed oxygen in ml to Joules relative to combustion enthalpy of substrates resulting from oxidation observed indirectly from respiratory exchange ratio [42]. The optimal walking speed was calculated fitting the individual subjects’ 2nd-order curvilinear regressions to the *C* values. We found the minimum value of the regressions (*C* = a speed^2^ + b speed + c) speed-cost algebraically by using the formula *x* = −*b*/2*a*. The oxygen consumption was also calculated and expressed in ml kg^−1^ min^−1^.

### Training

The training sessions were performed on an outdoor track. The duration of the training program was 8 weeks, with the frequency of three weekly sessions. The sessions were organized in the initial part or warming-up, the main part, and the final part or cooling-down, totalling 60 min each training session. The volume and intensity during the training period are described in Table 1. The volume (session duration in minutes) and intensity (percent heart rate associated with second ventilatory threshold reached during the session) were the same for both groups, only differing in the use (NW) and non-use (FW) of the poles during the sessions of walking. The load distribution was, in general, undulating and progressive, with intensities ranging from 70 to 105% of the heart rate associated with the second ventilatory threshold, determined from maximal tests. The average heart rate associated with the second ventilatory threshold was 129 bpm at baseline; therefore, the intensities ranged, on average, from 90 to 136 bpm, respectively, to 70 and 105%. We reported the intervention description in Template for Intervention Description and Replication (TIDieR) checklist (Additional file 1). Heart rate was controlled by the cardiac monitor during all training sessions. Trained NW instructors attended all training sessions. The dataset is in Additional file 2.

**Table 1:** Nordic walking and free walking training periodization, with 24 sessions over 8 weeks. Initial part **(IP)**, main part **(MP),** and final part **(FP)** refer to the training session. The different training intensities were light rhythm (~ 70% of 2VT), moderate (~ 80% 2VT), or strong rhythm (from 80 to 105% of 2VT) and was verbally indicated to the participants, during the main part of the training session. The volume of the training session was given in minutes. Interval training is indicated by the recovery time in the micro-pause in each session

Macrocycle		
**Microcycle I**		
**Session 1**	**Session 2**	**Session 3**
**IP:** 5′ 70% **MP:** 2 × 10′, 80% with 2′ of recovery **FP:** 5′ 70%	**IP:** 5′ 70% **MP:** 5× (4′ 85% + 1′ 75%) **FP:**5′ 75%	**IP:** 10′ 70% **MP:** 25′ 90% **FP:** 5′ 70%
**Session 4**	**Session 5**	**Session 6**
**IP:** 5′ 75% **MP:** 5× (3′ 95% + 2′ 70%) **FP:** 5′ 75%	**IP:** 10′ 70% **MP:** 2× (8′ 80% + 2′ 95%) **FP:** 5′ 70%	**IP:** 5′ 75% **MP:** 3× (5′ 90% + 5′ 80%) **FP:** 5′ 75%
**Session 7**	**Session 8**	**Session 9**
**IP:** 5′ 70% **MP:** 2× (10′ 95% + 5′ 85%) with 2′ of recovery **FP:** 5′ 70%	**IP:** 10′ 80% **MP:** 5× (4′ 95% + 1′ 80%) **FP:** 5′ 75%	**IP:** 5′ 70% **MP:** 2× (20′ 85%) 2 with 2′ of recovery **FP:** 5′ 75%
**Session 10**	**Session 11**	**Session 12**
**IP:** 10′ 70% **MP:** 2× (10′ 95% + 10′ 85%) with 2′ of recovery **FP:** 5′ 70%	**IP:** 10′ 80% **MP:** 2× (15′ 85% + 5′ 70%) **FP:** 10′ 70%	**IP:** 5′ 70% **MP:** 2× (20′ 80% + 5′ 95%) with 2′ of recovery **FP:** 5′ 75%
**Microcycle II**		
**Session 13**	**Session 14**	**Session 15**
**IP:** 5′ 80% **MP:** 5× (3′ 100% + 2′ 75%) **FP:** 5′ 75%	**IP:** 5′ 70% **MP:** 6× (2′ 105% + 3′ 70%) **FP:** 10′ 80%	**IP:** 10′ 80% **MP:** 3× (10′ 95%) with 2′ of recovery **FP:** 10′ 75%
**Session 16**	**Session 17**	**Session 18**
**IP:** 10′ 70% **MP:** 5× (3′ 80% + 2′ 70%) **FP:** 5′ 70%	**IP:** 10′ 70% **MP:** 2× (15′ 85%) with 2′ of recovery **FP:** 5′ 70%	**IP:** 5′ 75% **MP:** 3× (10′ 85%) with 3′ of recovery **FP:** 5′ 75%
**Session 19**	**Session 20**	**Session 21**
**IP:** 5′ 75% **MP:** 3× (10´ 90% + 5´ 75%) **FP:** 5´ 70%	**IP:** 5′ 70% **MP:** 6× (4′ 90% + 1′ 100%) with 2′ of recovery **FP:** 10′ 80%	**IP:** 10′ 75% **MP:** 2× (15′ 95%) with 3′ of recovery **FP:** 1× (5′ 80% + 5′ 70%)
**Session 22**	**Session 22**	**Session 24**
**IP:** 10′ 80% **MP:** 5× (3′ 100% + 2′ 85%) with 2′of recovery **FP:** 10′ 80%	**IP:** 10′ 75% **MP:** 3× (10′ 95%) with 2′ of recovery **FP:** 10′ 75%	**IP:** 5′ 80% **MP:** 3× (5′ 85% + 5′ 105%) **FP:** 10′ 70%

### Statistical Procedures

The results were presented using descriptive analysis (mean ± and confidence interval). In the baseline, the sample characterization data of both groups (NW and FW) were compared using independent *t* test (for scalar variables) and Q-square test (for categorical variables). Two groups × 2 (pre-post) generalized estimating equations (GEE) and the Bonferroni post hoc test were used to compare means of all dependent variables (in the intention-to-treat analysis). In summary, the GEE analysis allows robustly estimate variances of the regression coefficient for data exhibiting a high correlation between repeated measurements, behaves robustly against outliers so provides consistent estimators, allows intention-to-treat analysis, works with missing data instead of repeated measures where the missing data cancel the subject. Then, the effect size (by Cohen’s d Method) was calculated using post-training values between NW and FW groups and were classified as small effect (between 0.2 and 0.5), moderate (between 0.5 and 0.8) and large effect (0.8 or more) [43], and those results are presented by mean and confidence interval of 95%. Significance level adopted was *α* = 0.05 and *α* = 0.01 for all tests. Statistical processing was done using SPSS software (Statistical Package for Social Sciences for Mac, version 22.0).

## Results

### Bioenergetics and Biomechanics

The flowchart of participants through the study is in Fig. 2. The elderly who finished the intervention demonstrated adherence to training, fulfilling an average frequency of 93%, ranging from 87 to 95%.

The analysis of the sample characteristics did not show differences between the training groups, as described in Table 2.

**Table 2:** Sample characterization (ITT analysis), presenting mean and confidence interval (95% CI) for age, height, body mass (BM), body mass index (BMI), waist/height ratio (WAI/HEI), sum of skinfolds (ΣSK), lower limb length (LLL) of both groups

Variable	FW group (*n* = 16)	NW group (*n* = 16)	*p* value
	Mean (95% CI)	Mean (95% CI)	
Age (years)	68 (66 to 70)	64 (62 to 66)	*0.006*
Height (m)	1.62 (1.57 to 1.67)	1.65 (1.62 to 1.69)	0.237
BM (kg)	74 (66 to 81)	81 (74 to 85)	0.210
Fat Percentage (%)	31 (27 to 35)	32 (27 to 37)	0.918
BMI (kg m^−2^)	28 (26 to 30)	29 (27 to 31)	0.694
WAI/HEI	0.87 (0.82 to 0.90)	0.91 (0.84 to 0.93)	0.472
ΣSK (mm)	132 (113 to 134)	132 (108 to 146)	0.740
LLL_right_ (m)	0.83 (0.80 to 0.86)	0.85 (0.83 to 0.88)	0.301
LLL_left_ (m)	0.83 (0.80 to 0.86)	0.86 (0.83 to 0.89)	0.175
Men, n (%)	4 (44.4)	5 (55.6)	0.686

Note: the groups were different at *p* < 0.05

The heart rate during submaximal walking tests on the treadmill was reduced from pre- to post-training (*x*^2^ = 5.82, *p* = 0.016) and increased with the increment of the walking speed (*x*^2^ = 243.96, *p* < 0.001) in both groups, but without significant interactions between the factors (Table 3).

**Table 3:** Results of the HR_exercise_, the *RPE* scale, and the *C* during exercise, presented in mean and SE. Different lower case letters represent significant differences between speeds. € symbol represents significant differences in factor time (pre- and post-training) within each group. *Significant differences between NW and FW groups (in pre- and post-training moments)

Variable	Speed	FW pre (mean ± SE)	FW post (mean ± SE)	FW pre (mean ± SE)	FW post (mean ± SE)	Group	Time	Speed	Group*time	Group*speed	Time*speed	Group*time*speed
						*p*						
HR_exercise_ (bpm)	1 km h^−1^	^€^85.25 ± 3.09^a^	80.44 ± 2.35^a^	^€^83.45 ± 3.85^a^	80.47 ± 3.80^a^	0.573	*0.016*	*< 0.001*	0.443	0.659	0.525	0.699
	2 km h^−1^	^€^90.26 ± 3.03^b^	84.87 ± 2.88^b^	^€^85.79 ± 3.53^b^	84.26 ± 3.77^a^							
	3 km h^−1^	^€^92.65 ± 3.16^b^	87.88 ± 2.99 ^b^	^€^89.25 ± 3.48^c^	87.53 ± 3.79^b^							
	4 km h^−1^	^€^99.05 ± 2.95^c^	92.58 ± 3.04 ^c^	^€^94.13 ± 3.78^d^	91.30 ± 3.65^c^							
	5 km h^−1^	^€^107.97 ± 4.03^d^	102.65 ± 3.67^d^	^€^103.68 ± 4.55^e^	98.86 ± 3.72^d^							
*RPE*	1 km h^−1^	^€^1.71 ± 0.23^a*^	1.50 ± 0.16^a*^	^€^1.33 ± 0.12^a*^	1.08 ± 0.08^a*^	*0.001*	*0.002*	*< 0.001*	0.280	*0.035*	*0.022*	*0.038*
	2 km h^−1^	1.93 ± 0.18^a*^	1.93 ± 0.18^a*^	^€^1.33 ± 0.12^a*^	1.17 ± 0.10^a*^							
	3 km h^−1^	^€^2.07 ± 0.18^a*^	1.86 ± 0.13^a*^	^€^1.67 ± 0.18^ab*^	1.42 ± 0.14^a*^							
	4 km h^−1^	^€^2.71 ± 0.15^b*^	2.14 ± 0.13^b*^	^€^2.07 ± 0.14^b*^	2.00 ± 0.11^b*^							
	5 km h^−1^	^€^3.79 ± 0.23^c*^	2.86 ± 0.17^c*^	^€^2.80 ± 0.19^c*^	2.58 ± 0.14^c*^							
*C* (J kg^−1^ m^−1^)	1 km h^−1^	8.83 ± 0.50^a^	7.72 ± 0.64^a^	8.07 ± 0.58^a^	6.55 ± 0.69^a^	0.056	*0.014*	*< 0.001*	0.261	0.536	*0.029*	0.256
	2 km h^−1^	5.60 ± 0.26^bc^	5.59 ± 0.37^bc^	5.44 ± 0.41^bc^	4.27 ± 0.28^bc^							
	3 km h^−1^	4.65 ± 0.25^bd^	4.44 ± 0.26^bd^	4.45 ± 0.29^bd^	3.68 ± 0.31^bd^							
	4 km h^−1^	4.54 ± 0.20^b^	4.36 ± 0.22^b^	4.18 ± 0.24^b^	3.76 ± 0.27^b^							
	5 km h^−1^	4.78 ± 0.21^b^	4.66 ± 0.22^b^	4.34 ± 0.27^b^	3.86 ± 0.25^b^							

HR_exercise_ represents exercise heart rate, *RPE* represents the result of the rating of perceived exertion scale, and *C* represents the cost of transport value during walking on the treadmill tests at different speeds. *p* represents the results of the statistical tests

The rating of perceived exertion during exercise was affected by group factor, showing lower values in the NW group than in the FW group. These values decreased from pre- to post-training moments (time factor) in both groups and by the speed (speed factor), increasing with the increment of walking speed in both groups. The factor interactions were significant as follows: group*speed (*x*^2^ 6.24, *p* = 0.035), time*speed (*x*^2^ 2.00, *p* = 0.022) and group*time*speed (*x*^2^ 132.3, *p* = 0.038), as shown in Table 3.

The *C* was reduced after the training (*x*^2^ 4.91, *p* = 0.014), irrespective of the group whereas remains unchanged (*x*^2^ 3.44, *p* = 0.056) for the group factor and the speed factor was significant (*x*^2^ 203.5, *p* < 0.001). The interaction between main factors was significant only in the time*speed analysis (*x*^2^ 8.45, *p* = 0.029), occurring a greater reduction from pre- to post-training in the *C* at slower speeds (1 km h^−1^ and 2 km h^−1^) than at higher speeds (4 km h^−1^ and 5 km h^−1^), see Table 3 and Fig. 4. The optimal walking speed was affected by time (*x*^2^ 8.94, *p* = 0.003), increasing from 3.50 ± 0.22 to 4.04 ± 0.14 km h^−1^ in FW group and from 3.81 ± 0.25 to 4.29 ± 0.25 km h^−1^ in NW group.

**Fig. 3:**
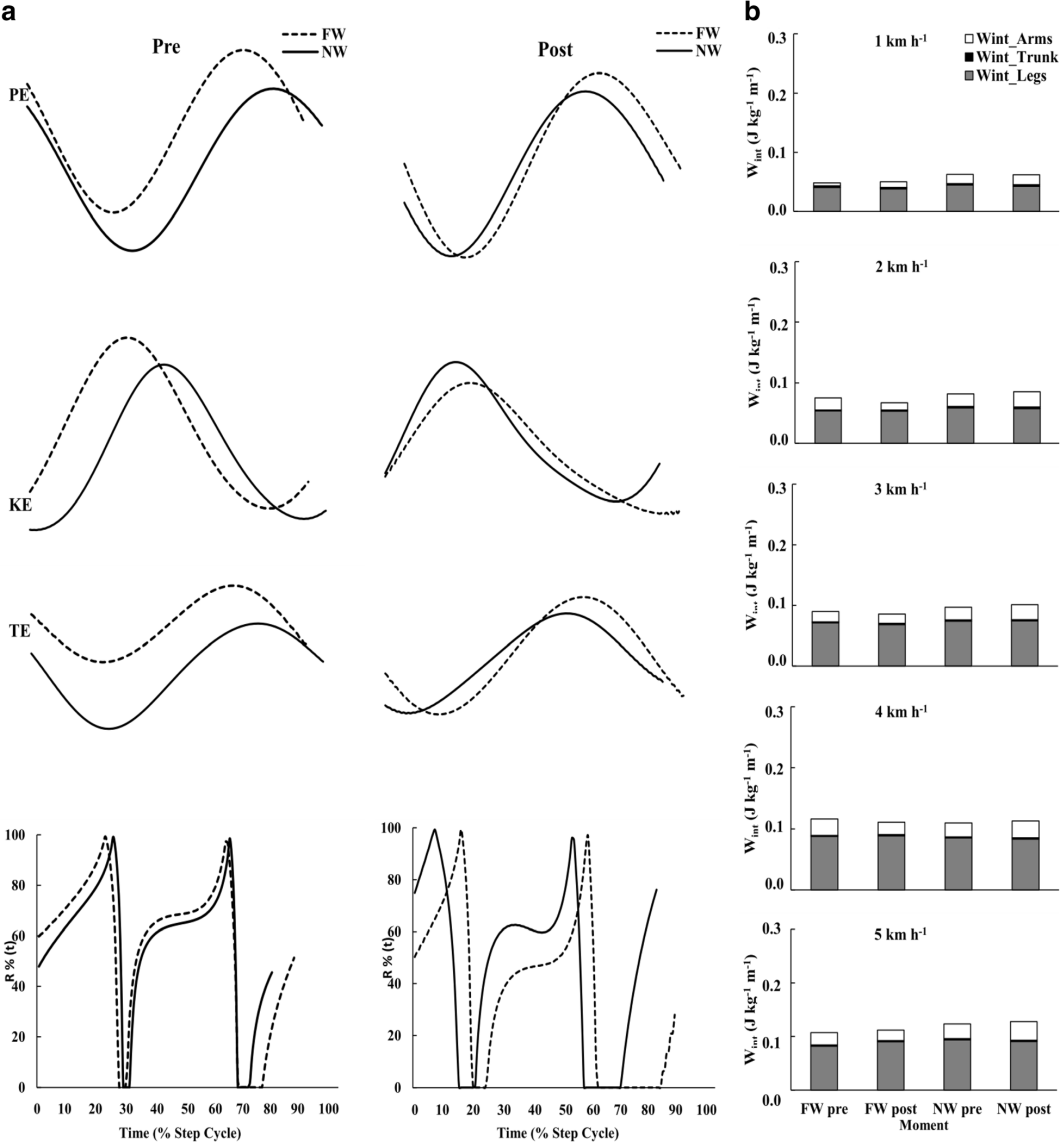
The left column is the example of the behavior of BCoM energies during treadmill walking (without poles) at 3 km h^−1^, at the pre- and post-training moments. Top panel PE and KE, and TE = PE + KE, lower panel *R*%(*t*) indicating the conversion percentage of the pendulum mechanism during the step time cycle. Divided lines FW group and continuous lines NW group, left and right side of the figure of the BCoM energies represent the pre- and post-training moment, respectively. In the right column are the results of the Wint_legs, Wint_trunk, and Wint_arms of FW and NW groups in pre- and post-training moments, in the different speeds (results are in mean values, for more information see Table 4)

Table 4 shows the mechanical work variables. The *W*_ext_ demonstrated a significant group*time*speed interaction (*x*^2^ 13.37, *p* = 0.010), presenting a reduced *W*_ext_ after FW and maintenance in the group NW. Also, the mean values of PE, KE, total external energy, and *W*_int_ are observed in Fig. 3.

**Table 4:** Results from GEE model effect tests for mechanical work variables. Results presented by mean and SE. Different low case letters represent that there were difference between speeds. * symbol represents that there was difference between NW and FW groups in the different moments. € symbol represents that there were differences between pre- and post moments within each group.

Variable	Speed	FW pre (mean ± SE)	FW post (mean ± SE	FW pre (mean± SE)	FW post (mean ± SE)	Group	Time	Speed	Group*time	Group*speed	Time*speed	Group*time*speed
						*p*						
W_ext_ (J kg^−1^ m^−1^)	1 km h^−1^	0.192 ± 0.12^a^	*0.193 ± 0.12^a^	0.188 ± 0.18^a^	*0.176 ± 0.13^a^	0.354	0.837	< 0.001	0.049	0.950	0.259	0.010
	2 km h^−1^	0.212 ± 0.11^a^	*0.196 ± 0.07^a^	0.215 ± 0.04^a^	*0.220 ± 0.18^a^							
	3 km h^−1^	0.216 ± 0.12^a^	*0.205 ± 0.13^a^	0.216 ± 0.11^a^	*0.216 ± 0.08^a^							
	4 km h^−1^	0.208 ± 0.16^a^	*0.178 ± 0.11^a^	0.195 ± 0.10^a^	*0.201 ± 0.07^a^							
	5 km h^−1^	0.181 ± 0.18^a^	*0.151 ± 0.15^b^	0.160 ± 0.14^b^	*0.155 ± 0.13^b^							
*W*_int_ (J kg^−1^ m^−1^)	1 km h^−1^	0.048 ± 0.04^a^	0.050 ± 0.04^a^	0.062 ± 0.04^a^	0.062 ± 0.04^a^	0.234	0.383	*< 0.001*	0.383	0.421	0.385	0.579
	2 km h^−1^	0.075 ± 0.04^a^	0.067 ± 0.06^a^	0.081 ± 0.05^bc^	0.085 ± 0.01^b^							
	3 km h^−1^	0.089 ± 0.05^b^	0.085 ± 0.06^c^	0.096 ± 0.04^c^	0.101 ± 0.04^c^							
	4 km h^−1^	0.116 ± 0.04^c^	0.110 ± 0.05^d^	0.109 ± 0.01^d^	0.113 ± 0.06^d^							
	5 km h^−1^	0.117 ± 0.01^c^	0.111 ± 0.05^d^	0.123 ± 0.05^e^	0.122 ± 0.05^d^							
*W*_tot_ (J kg^−1^ m ^−1^)	1 km h^−1^	0.268 ± 0.15^a^	0.258 ± 0.13^a^	0.275 ± 0.21^a^	0.254 ± 0.16^a^	0.233	0.384	*< 0.001*	0.382	0.582	0.333	0.390
	2 km h^−1^	0.309 ± 0.13^b^	0.310 ± 0.11^b^	0.303 ± 0.07^a^	0.329 ± 0.28^ab^							
	3 km h^−1^	0.340 ± 0.12^b^	0.490 ± 0.63^bc^	0.339 ± 0.12^a^	0.347 ± 0.07^b^							
	4 km h^−1^	0.459 ± 0.44^c^	0.367 ± 0.13^b^	0.349 ± 0.19^ab^	0.382 ± 0.09^bc^							
	5 km h^−1^	0.404 ± 0.13^bc^	0.386 ± 0.95^b^	0.386 ± 0.16^b^	0.391 ± 0.14^c^							
*W*_int_trunk_ (J kg^−1^ m^−1^)	1 km h^−1^	0.0027 ± 0.001^a^	0.0024 ± 0.001^a^	0.0026 ± 0.002^a^	0.0026 ± 0.001^ab^	0.217	0.204	*< 0.001*	0.329	0.301	0.547	*0.024*
	2 km h^−1^	0.0017 ± 0.001^a^	0.0018 ± 0.001^b^	0.0022 ± 0.001^ab^	0.0027 ± 0.002^ab^							
	3 km h^−1^	0.0015 ± 0.001^ab^	0.0021 ± 0.002^ab^	0.0021 ± 0.003^ab^	0.0021 ± 0.001^a^							
	4 km h^−1^	0.0016 ± 0.002^ab^	0.0019 ± 0.001^b^	0.0017 ± 0.001^c^	0.0020 ± 0.001^a^							
	5 km h^−1^	0.0022 ± 0.001^a^	0.0022 ± 0.001^ab^	0.0023 ± 0.001^a^	0.0024 ± 0.001^b^							
*W*_int_arms_ (J kg^−1^ m^−1^)	1 km h^−1^	^€^0.005 ± 0.071^a^	*0.009 ± 0.019^a^	^€^0.015 ± 0.030^a^	*0.017 ± 0.013^a^	0.181	0.865	*< 0.001*	*0.032*	*0.023*	0.436	0.874
	2 km h^−1^	^€^0.019 ± 0.038^b^	*0.012 ± 0.046^a^	^€^0.020 ± 0.037^b^	*0.025 ± 0.070^ab^							
	3 km h^−1^	^€^0.017 ± 0.030^b^	*0.015 ± 0.033^a^	^€^0.021 ± 0.036^b^	*0.024 ± 0.052^ab^							
	4 km h^−1^	^€^0.027 ± 0.049^c^	*0.020 ± 0.036^b^	^€^0.022 ± 0.040^b^	*0.027 ± 0.052^ab^							
	5 km h^−1^	^€^0.023 ± 0.032^c^	*0.019 ± 0.027^b^	^€^0.027 ± 0.045^c^	*0.034 ± 0.058^c^							
*W*_int_legs_ (J kg^−1^ m^−1^)	1 km h^−1^	0.040 ± 0.019^a^	0.038 ± 0.021^a^	0.044 ± 0.021^a^	0.043 ± 0.020^a^	0.234	0.384	< 0.001	0.383	0.536	0.468	0.469
	2 km h^−1^	0.053 ± 0.023^b^	0.053 ± 0.029^b^	0.058 ± 0.020^b^	0.057 ± 0.054^b^							
	3 km h^−1^	0.071 ± 0.021^c^	0.068 ± 0.036^c^	0.073 ± 0.019^c^	0.074 ± 0.015^c^							
	4 km h^−1^	0.087 ± 0.025^d^	0.088 ± 0.024^d^	0.085 ± 0.065^d^	0.083 ± 0.021^d^							
	5 km h^−1^	0.081 ± 0.063^d^	0.089 ± 0.024^d^	0.093 ± 0.023^e^	0.090 ± 0.024^e^							
*R* (%)	1 km h^−1^	32.46 ± 3.07^a^	35.40 ± 2.45^a^	33.5 9 ± 4.07^a^	34.89 ± 4.41^a^	0.288	0.085	*< 0.001*	0.267	0.702	0.065	0.671
	2 km h^−1^	37.85 ± 2.26^a^	35.54 ± 2.38^a^	40.45 ± 3.36^a^	39.73 ± 4.02^a^							
	3 km h^−1^	45.26 ± 3.44^b^	46.95 ± 2.42^b^	49.08 ± 2.73^bc^	48.72 ± 3.05^bc^							
	4 km h^−1^	50.39 ± 3.42^b^	56.83 ± 2.70^c^	58.07 ± 3.35^d^	59.16 ± 2.10^d^							
	5 km h^−1^	63.97 ± 4.45^c^	69.57 ± 1.62^d^	70.65 ± 2.68^e^	72.43 ± 2.46^e^

The *W*_int_ was only affected by walking speed (*x*^2^ 1360.30, *p* < 0.001), increasing with the increment of the speed (Table 4). The interactions were not significant. However, *W*_int_trunk_ was affected by group*time*speed (*x*^2^ 258.14, *p* = 0.024; Table 4 and Fig. 3). Similarly, *W*_int_legs_ was only affected by walking speed (*x*^2^ 1399.73, *p* < 0.001) increasing with the increment of the speed in both groups (Table 4 and Fig. 3), and the interactions were not significant. Nevertheless, by analyzing *W*_int_arms_, it was affected by walking speed (*x*^2^ 26.18, *p* < 0.001), increasing with the increment of the speed, and also by group*time interaction (*x*^2^ 4.318, *p* = 0.032), representing a greater *W*_int_arms_ in NW group after training when compared with FW group, and in group*speed interaction (*x*^2^ 13.21, *p* = 0.023), decreasing the values from pre to post-training in FW group and increasing the values from pre- to post-training in NW group (Table 4 and Fig. 3).

**Fig. 4:**
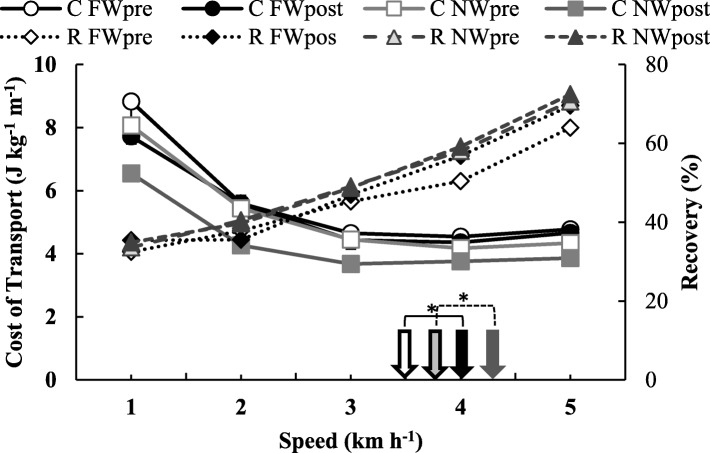
Result of the cost of transport (*C*, left vertical axis) and Recovery (*R*, right vertical axis) at different walking speeds. Results are presented as mean, white and black circles with continuous lines and light gray square and dark gray square with continuous lines represent *C* values from the FW group and NW group in pre- and post-training moments, respectively. White and black diamond with dotted lines and light gray and dark gray triangles with dotted lines represent *R* values from the FW group and NW group in pre-and post-training moments, respectively. The white and black arrows represent the optimal speed (OPT that is the speed at the minimal *C* of walking) of FW group (from pre- to post-training was from 3.50 to 4.04 km h^−1^ ), and the light gray and dark gray arrows represents the OPT of NW group (from pre- to post-training was from 3.81 to 4.29 km h^−1^). * Symbol between white and black arrows united by the continuous indicator and the * between light gray and dark gray arrows united by the indicator with dotted line represents significant difference (*p* < 0.05) in factor time in OPT of FW and NW group (from pre to post-training), respectively

The *W*_tot_ was only affected by walking speed (*x*^2^ 99.92, *p* < 0.001). The interactions were not significant. The *R* results demonstrated that there was a significant difference only in factor speed (*x*^2^ 296.68, *p* < 0.001), increasing concomitantly with speed. The values of the results of *C* and *R* can be observed in Fig. 4 (top and bottom panel, respectively) and also in Table 4.

### Neuromuscular Parameters

**Fig. 5:**
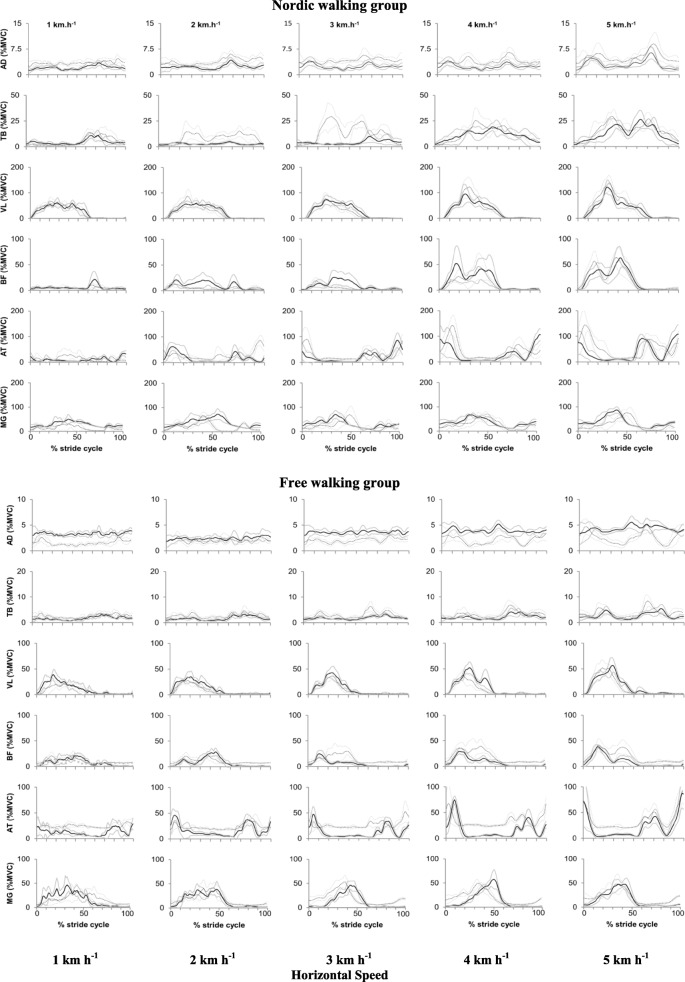
Example of the behavior of the mean amplitude of the signal normalized by the percentage of the stride throughout the different speeds of one subject from NW group (left side) and one subject from FW group (right side) in pre- and post-training moments (in continuous and dotted lines, respectively). Data presented in means (black lines) ± 1SD (gray lines). Note: AD, TB, VL, BF, AT and MG represents the anterior deltoid; the triceps brachii; the vastus lateralis; the biceps femoris; the anterior tibialis and the medial gastrocnemius muscles, respectively

Table 5 and Fig. 5 display the values of the EMG. The mean amplitude for the DA muscle was affected by group*time*speed (*x*^2^ 21.91, *p* = 0.021) interaction, with lower activation in the NW group when compared with FW in post-training, and also with a higher activation with increasing speed in both groups. TB muscle was only modified by walking speed (*x*^2^ 10.23, *p* = 0.020), increasing with the increment of the speed in both groups independently of the time.

**Table 5:** Results of mean amplitude of AD, TB, VL, BF, AT, and MG muscles in the different walking speeds presented in mean and SE, values in millivolts. Different lower case letters represent significant differences between speeds. *Significant differences between groups in pre- or post-training. ^€^Significant difference between pre- and post-training moment within each group

Variable	Speed	FW pre (mean ± SE)	FW post (mean ± SE)	NW pre (Média ± SE)	NW post (média ± SE)	Group	Time	Speed	Group*time	Group*speed	Time*speed	Group*time*speed
						*p*						
Mean amplitude AD	1 km h^−1^	^€^4.39 ± 0.66^a*^	6.26 ± 1.85^a*^	5.15 ± 1.02^a*^	5.49 ± 1.77^a*^	0.108	0.314	*0.003*	0.746	*0.006*	0.266	*0.021*
	2 km h^−1^	5.86 ± 1.80^a*^	5.49 ± 1.24^a*^	^€^2.89 ± 0.59^b*^	4.92 ± 1.43^a*^							
	3 km h^−1^	6.60 ± 2.03^a*^	6.63 ± 1.61^a*^	^€^3.90 ± 0.84^b*^	5.68 ± 1.67^a*^							
	4 km h^−1^	^€^5.09 ± 1.14^a*^	8.63 ± 2.17^a*^	3.54 ± 0.69^b*^	5.39 ± 1.89^a*^							
	5 km h^−1^	^€^7.85 ± 1.72^b*^	9.78 ± 2.21^b*^	^€^3.44 ± 0.53^b*^	5.72 ± 1.70^a*^							
Mean amplitude TB	1 km h^−1^	3.87 ± 0.59^a^	6.15 ± 1.48^a^	6.91 ± 2.13^a^	4.80 ± 1.23^a^	0.614	0.967	*0.020*	0.948	0.498	0.193	0.083
	2 km h^−1^	5.37 ± 1.16^a^	7.90 ± 3.04^a^	7.19 ± 2.99^a^	6.70 ± 1.75^b^							
	3 km h^−1^	8.35 ± 2.82^b^	9.81 ± 3.49^b^	6.47 ± 2.55^a^	7.99 ± 1.61^b^							
	4 km h^−1^	11.67 ± 4.02^c^	9.11 ± 3.13^b^	7.65 ± 3.76^a^	8.65 ± 1.76^bc^							
	5 km h^−1^	14.15 ± 4.15^c^	9.46 ± 2.73^b^	8.10 ± 2.18^b^	8.10 ± 1.80^b^							
Mean amplitude VL	1 km h^−1^	6.12 ± 0.95^a^	6.21 ± 0.79^a^	8.75 ± 2.84^a^	8.52 ± 2.30^a^	0.425	0.302	*< 0.001*	0.352	0.427	0.619	0.330
	2 km h^−1^	7.17 ± 1.56^a^	7.45 ± 0.92^a^	8.10 ± 1.88^a^	9.20 ± 2.29^a^							
	3 km h^−1^	9.27 ± 2.19^a^	8.53 ± 1.04^a^	10.19 ± 2.34^b^	13.7 ± 3.70^b^							
	4 km h^−1^	13.20 ± 2.28^b^	12.84 ± 1.65^b^	10.54 ± 2.06^b^	17.3 ± 4.07^b^							
	5 km h^−1^	14.36 ± 2.75^b^	15.87 ± 1.81^b^	15.51 ± 3.61^c^	20.4 ± 5.37^c^							
Mean amplitude BF	1 km h^−1^	7.87 ± 2.07^a*^	11.20 ± 3.01^a*^	5.99 ± 1.18^a*^	4.19 ± 1.23^a*^	*0.045*	0.645	*< 0.001*	0.791	0.270	0.746	0.620
	2 km h^−1^	10.67 ± 2.47^a*^	10.42 ± 1.54^a*^	7.14 ± 1.45^a*^	5.60 ± 1.62^a*^							
	3 km h^−1^	11.10 ± 2.40^a*^	10.91 ± 1.70^a*^	10.15 ± 3.04^b*^	7.32 ± 2.23^b*^							
	4 km h^−1^	15.46 ± 3.87^b*^	14.16 ± 1.95^b*^	9.74 ± 1.66^b*^	9.13 ± 2.67^b*^							
	5 km h^−1^	21.11 ± 5.91^c*^	17.54 ± 2.59^c*^	12.03 ± 1.86^c*^	11.50 ± 3.27^c*^							
Mean amplitude AT	1 km h^−1^	12.53 ± 2.97^a^	14.20 ± 2.62^a^	12.24 ± 1.56^a^	9.67 ± 2.35	0.127	0.228	*< 0.001*	0.382	0.082	0.439	0.764
	2 km h^−1^	14.33 ± 2.98^a^	16.91 ± 3.93^b^	17.52 ± 2.20^b^	13.16 ± 3.33							
	3 km h^−1^	16.54 ± 3.23^b^	17.86 ± 2.84^b^	19.27 ± 2.93^b^	14.21 ± 3.32							
	4 km h^−1^	21.95 ± 4.20^c^	22.11 ± 3.20^c^	24.34 ± 3.12^c^	17.38 ± 4.12							
	5 km h^−1^	28.97 ± 5.87^d^	28.16 ± 3.93^d^	31.37 ± 4.04^d^	23.73 ± 5.24							
Mean amplitude MG	1 km h^−1^	11.94 ± 2.23^a^	12.91 ± 2.10^a^	15.06 ± 2.42^a^	10.40 ± 2.48^a^	0.196	0.391	*< 0.001*	0.463	0.627	0.058	0.179
	2 km h^−1^	12.85 ± 2.49^a^	15.65 ± 3.16^b^	16.13 ± 2.50^a^	13.08 ± 3.19^b^							
	3 km h^−1^	15.27 ± 3.37^b^	15.59 ± 2.27^b^	16.19 ± 2.73^a^	14.51 ± 3.34^b^							
	4 km h^−1^	16.89 ± 3.16^b^	19.16 ± 2.77^c^	20.19 ± 2.93^b^	14.89 ± 3.46^b^							
	5 km h^−1^	20.62 ± 4.70^c^	19.99 ± 2.89^c^	22.28 ± 3.16^b^	16.77 ± 3.63^c^

Concerning leg muscles, VL, BF, TA, and GM muscles were affected by walking speed (*x*^2^ 67.83; *x*^2^ 53.66; *x*^2^ 78.57; *x*^2^ 60.13 respectively and *p* < 0.001 for all) increasing with the increment of the speed in both groups, independently of the time. The activation of the BF muscle was higher in the FW group than that in the NW group, independently of the speed and time, as demonstrated in Table 5.

The co-contraction results of DA/TB demonstrated that there was an only modification in factor time (*x*^2^ 5.24, *p* = 0.022), decreasing the co-contraction value from pre to post-training in both groups (Table 6). Co-contraction of VL/BF muscles demonstrated that these legs proximal muscles were not affected by speed, time, or group (Table 6). About the co-contraction of TA/GM, it demonstrated that these muscles were affected by group*speed interaction (*x*^2^ 42.44, *p* = 0.012), decreased the value of the co-contraction of TA/GM with the increasing speed in both groups. Also, the TA/GM co-contraction was lower in the NW group than that in the FW group irrespective of speed and time as observed in Table 6.

**Table 6:** Co-contraction results of AD/TB, VL/BF, and AT/MG respectively, in the different walking speeds. Values presented by mean and SE. *Significant differences in pre- and post-training moments between groups. Different lower case letters represent significant differences between speeds. ^€^Significant differences between pre- and post-training moments of each group

Variable	Speed	FW pre (mean ± SE)	FW post (mean ± SE)	NW pre (média ± SE)	NW post (média ± SE)	Group	Time	Speed	Group*time	Group*speed	Time*speed	Group*time*speed
						*p*						
Co-Contraction AD/TB	1 km h^−1^	^€^52.01 ± 7.21	49.33 ± 5.20	^€^60.36 ± 5.91	47.06 ± 7.69	0.600	*0.022*	0.680	0.120	0.218	0.716	0.591
	2 km h^−1^	^€^56.28 ± 6.53	50.45 ± 5.31	^€^54.40 ± 5.25	45.28 ± 4.22							
	3 km h^−1^	^€^53.99 ± 4.58	48.66 ± 5.39	^€^54.96 ± 5.78	34.77 ± 6.19							
	4 km h^−1^	^€^48.81 ± 5.06	51.07 ± 4.89	^€^58.01 ± 4.06	42.84 ± 5.58							
	5 km h^−1^	^€^53.86 ± 6.37	51.07 ± 4.57	^€^54.92 ± 4.70	37.39 ± 3.66							
Co-Contraction VL/BF	1 km h^−1^	46.66 ± 4.21	45.63 ± 4.41	45.05 ± 4.46	48.91 ± 5.96	0.502	0.939	0.244	0.991	0.402	0.114	0.527
	2 km h^−1^	40.35 ± 4.27	44.55 ± 3.88	46.66 ± 3.53	50.37 ± 5.04							
	3 km h^−1^	42.36 ± 4.76	45.34 ± 4.53	47.17 ± 2.78	47.06 ± 3.93							
	4 km h^−1^	48.94 ± 3.04	48.37 ± 4.73	51.74 ± 1.48	48.68 ± 3.13							
	5 km h^−1^	50.11 ± 2.68	45.94 ± 3.98	49.55 ± 2.83	46.19 ± 3.65							
Co-Contraction AT/MG	1 kmh^−1^	38.05 ± 2.91^a*^	45.00 ± 4.26^a*^	32.99 ± 1.76^a*^	39.81 ± 2.63^a*^	0.169	0.365	*< 0.001*	0.880	*0.012*	0.649	0.436
	2 km h^−1^	36.55 ± 4.11^a*^	39.06 ± 3.76^b*^	30.72 ± 1.57^a*^	34.13 ± 1.93^ab*^							
	3 km h^−1^	33.90 ± 4.32^a*^	35.95 ± 2.70^b*^	30.68 ± 1.44^a*^	31.75 ± 2.36^b*^							
	4 km h^−1^	33.64 ± 4.08^a*^	34.07 ± 2.69^b*^	30.46 ± 2.22^a*^	31.42 ± 2.06^b*^							
	5 km h^−1^	30.99 ± 4.16^b*^	28.35 ± 2.80^c*^	27.48 ± 2.11^a*^	28.22 ± 1.68^b*^							

Still, the results of the mean amplitude of the signal time normalized by the percentage of the strides in the different speeds of pre- and post-training moments from a subject from the NW group with a subject from the FW group are described in Fig. 5.

**Fig. 6:**
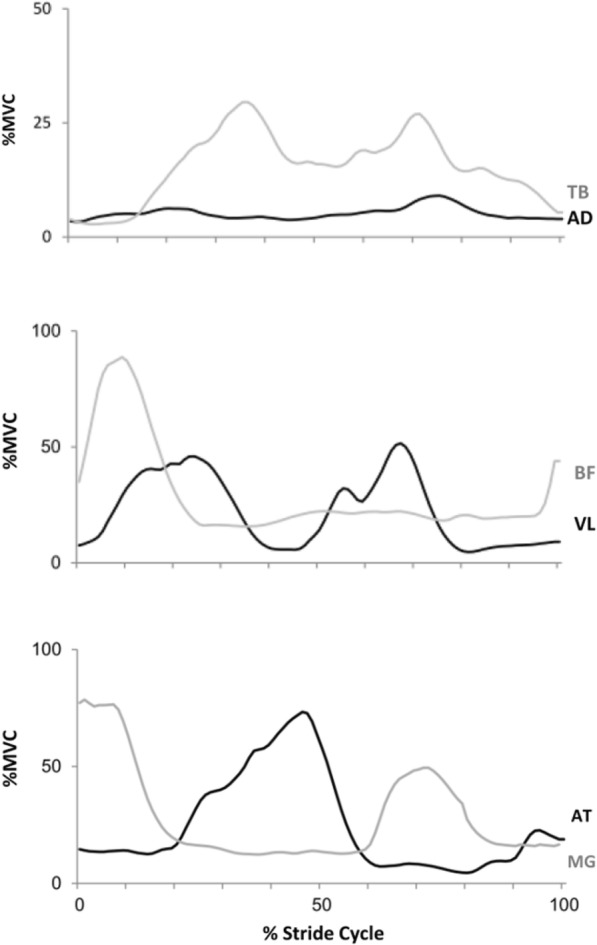
Representative curves for one subject (NW group at post-training) of the muscular co-contraction analysis. The co-contraction is the overlap area on antagonist muscles

The representative curves of the co-contraction routine are described in Fig. 6.

Also, the summary of the findings regarding energetics and mechanics parameters and EMG parameters are presented in Table 7.

**Table 7:** The summary of the variables analyzed in the different factors and interactions

Variable	Group	Time	Speed	Group*time	Group*speed	Time*speed	Group*time*speed
	*p*						
HR_exercise_	=	↓	↑	0.443	0.659	0.525	0.699
RPE	↑	↓	↑	0.280	*0.035*	*0.022*	*0.038*
*C*	=	=	↓	0.261	0.536	0.029	0.256
*W*_ext_	↑	=	↑↓	*0.049*	0.950	0.259	0.721
*W*_int_	=	=	↑	0.383	0.421	0.385	0.579
*W*_tot_	=	=	↑	0.382	0.582	0.333	0.390
*W*_int_Trunk_	=	=	↓↑	0.329	0.301	0.547	*0.024*
*W*_int_Legs_	=	=	↑	0.383	0.536	0.468	0.469
*W*_int_Arms_	=	=	↑	*0.032*	*0.023*	0.436	0.874
*R* (%)	=	=	↑	0.267	0.702	0.065	0.671
Mean_Amp AD	↓	↑	↑	0.746	*0.006*	0.266	*0.021*
Mean_Amp TB	=	=	↑	0.948	0.498	0.193	0.083
Mean_Amp VL	=	=	↑	0.352	0.427	0.619	0.330
Mean_Amp BF	↓	=	↑	0.791	0.270	0.746	0.620
Mean_Amp AT	=	=	↑	0.382	0.082	0.439	0.764
Mean_Amp MG	=	=	↑	0.463	0.627	0.058	0.179
C-C AD/TB	=	↓	=	0.120	0.218	0.716	0.591
C-C VL/BF	=	=	=	0.991	0.402	0.114	0.527
C-C AT/MG	=	=	↓	0.880	*0.012*	0.649	0.436

The arrows ↑ or ↓ in factor group represent that this variable was greater or lower, respectively, in NW group in comparison with FW group

## Discussion

To our knowledge, this is the first controlled and randomized trial reporting the effects of NW training and the adaptations in energetics and mechanics parameters and EMG at different walking speeds in sedentary elderly people. Our purpose was to compare the possible benefits of NW versus FW training on the *C*, mechanical work, muscular activation, and rating of perceived exertion of elderly people. The primary hypothesis of the study was rejected since *C* decreased similarly between NW and FW groups. Further, we expected a reduced co-contraction in lower limb muscles after training, due to the negative role of co-contraction on the metabolic economy in elderly based on a previous observational study [5]. The co-contraction from lower limb muscles remains unchanged in both groups. Probably, exercise programs directed toward improving the joint stability as strength and balance training seems to be possible candidates to decrease the muscle co-contraction in lower limbs, resulting in further enhancement of walking economy and functional mobility in elderly.

### Effects of NW Training in the Energetics and Biomechanics of Elderly People

It has been observed in observational studies that the heart rate and oxygen consumption are higher using poles whereas the rating of perceived exertion is similar [44–50]. The mismatch between the metabolic stress and perceptual response in NW in comparison with FW offers an exciting alternative for improving cardiorespiratory fitness in the health context. In our study, the exercise intensity was strictly controlled and individualized using percentages of anaerobic threshold (see Table 2), and therefore, we may claim that similar gains in both groups were obtained with lower perceptual stressors for the NW. Conversely, the intensity control based on perceptual responses used in previous studies, where the metabolic intensity was higher using poles, likely explains the higher adaptations from NW than FW training [47, 51]. Therefore, future studies may test how the mismatch between perceptual and metabolic loads in the NW should interfere with the prevention of potential imbalances in physical training as non-functional overreaching and overtraining. Individuals with Parkinson’s disease seem to be most sensitive to the usage of poles concerning functional mobility than elderly without neurodegenerative illness. Monteiro et al. [10] have shown that the dopaminergic nigrostriatal pathways are liberated using the technique of NW affecting the functional mobility positively in comparison to FW. Therefore, the NW training may be a useful alternative in aging groups with frailty and movement/cognitive disorders due to dual-task condition with increased cognitive demands.

Although the walking mechanics remains predominantly unchanged after both training programs, two interesting alterations in upper limbs need further consideration. The increased *W*_int_ to swing the upper limbs found in the NW group may be attributed to the transfer of movement pattern from NW to FW, where it has been well demonstrated the larger range of shoulder motion performed using poles than in normal walking [9]. Another finding regarding upper limbs was the reduction in shoulder muscles’ co-contraction in both groups, showing better effectiveness of upper limb movements for the NW group. This finding results in the optimization of dynamical stability and energy expenditure in human walking [51]. The EMG of DA was reduced after NW training while no differences for the FW group, reducing the *C*, in combination with improved dynamical stability [52]. Specific mechanisms regulating this transfer will be analyzed in future study.

The effects of different speeds on biomechanic and energetic responses in our study are in line with previous studies in adults [36, 53, 54] and even in elderly [5]. Interestingly, our study showed that speed-cost profiles were altered by endurance training. In fact, the reduction in *C* was most sensitive at slow speeds than higher speeds demonstrated by the significant time*speed interaction. Both training programs were able to increase the optimal walking speed by approximately 0.5 km h^−1^. This finding is in line with earlier observations in self-selected walking speed in elderly [52]. The self-selected speed is a feasible proxy for evaluating and monitoring daily ambulatory activity in elderly [54]. Therefore, both training programs were capable of increasing walking ability. This is the first finding in the literature showing an increase in optimal walking speed after endurance training. This novel finding may be attributed to better adaptation on the metabolic economy to walk at faster speeds analyzed here as a consequence of walking speeds used by our subjects during the interventions, between 5 and 7 km h^−1^. Specific adaptations in the economy at habitual training speeds have been found in running. The improvements in self-selected walking and optimal walking speeds have relevance for health promotion due to the inverse association between habitual walking speed and risk of falls [55].

These results do not corroborate the findings of Figueiredo et al. [8] who reported an increase in the self-selected walking speed of elderly people from the NW group in comparison to the FW group. The dissimilarity seems to be due to training loads used on studies. The elderly from Figueiredo et al. [8] trained with reduced training volume (twice a week, with duration of 20 min daily versus average session time of 60 min in our study) and the intensity of training was not controlled (comfortable rhythm versus 80–105% of the heart rate associated with second ventilatory threshold in our study). Therefore, it is known that NW in a comfortable rhythm is performed in higher exercise intensity and faster progression speed than FW [44, 48–50] justifying the differences between studies.

It is worth noting that gait mechanics especially related to leg movements were not altered after both training programs. And, the NW as well FW were able to improve functional mobility (as shown by Takeshima et al.) [6] confirming that after these programs elderly walk during their daily life activities with most optimized *R*, thus resulting in a most economical mobility due to improved motor (muscle and cardiorespiratory systems) and machine (pendulum-like mechanism) attributes [56].

Our results indicated that the mechanism of pendular energy reconversion (*R*) was not altered after both training programs and are in line with findings in adults [35, 36, 57], and in elderly [5] extending these findings to aerobically trained seniors. The neuromuscular adaptations of the elderly in response to NW and FW training demonstrated that a systematized walking training program (with and without poles) promotes positive neuromuscular adaptations in elderly people, therefore delaying the losses of the aging process. Essentially, our results of lower limbs muscular activation presented maintenance in group and time factors and group*time interaction, and such outcome was modified with the increment of the speed for the muscles: DA, VL, BF, TA, and GM, which was expected according to the literature [33].

It is known that muscle activation during locomotion results from the mechanisms involved in the generation of central patterns and sensory feedback, which certainly in elderly people is impaired by the aging [57]. These factors in combination with a sedentary lifestyle can result in lower muscle responses during the gait. On the other hand, a higher EMG during the gait can indicate a greater instability due to the deficits of motor control, mainly in sedentary elderly people [58]. In fact, it was found in the study of Mian et al. [5] where the co-activation of lower limbs muscles was correlated with the *C* and presented higher values in elderly when compared with young people. In the present study, the analysis method was more specific, based on the amplitude of the EMG and not only in the activation time. Our study demonstrates that in the thigh and shank musculature, the models of endurance training applied in the present study did not decrease co-contraction levels.

These findings associated with characteristics of careful training load control (volume and intensity) performed in the present study indicate that the central metabolic adaptations seem to have a determinant role in the adaptations found here. The NW is an effective training method in promoting locomotor adaptations arising from physical training in elderly people, showing that, when training volume and intensity are well controlled, the dose-response relationship is satisfactory.

## Conclusion

We conclude that NW is an effective training technique as FW for sedentary elderly people who require the development of cardiovascular endurance. The endurance walking training using or not using poles reduced the *C* of walking mainly due to contractile machinery and partly due to a reduction in the co-contraction activity for the shoulder muscles. Also, after the endurance training with and without poles, the elderly increase the optimal and self-selected walking speeds, and changed the speed-cost profile.

## Study Limitations

The training protocols were applied in an open environment and the evaluations were performed on a treadmill. Although there are minor differences between treadmill and floor walking, the treadmill has some advantages due to strict control of speed. Also, we believe that some specific adaptations would present better results if the tests were performed with the poles on the treadmill. Nevertheless, we choose testing the individuals without poles, in conditions closer to their daily life, with greater ecological validity from the health point of view.

## Supplementary information

**Additional file 1.** Template for Intervention Description and Replication (TIDieR).

**Additional file 2.** Dataset of the trained NW instructors who attended all training sessions.

## Data Availability

The dataset is in supplementary material 01.

## Abbreviations

BCoM: Body center of mass
BF: Biceps femoris muscle
*C*: Metabolic cost
C-C DA/TB: Co-contraction between deltoideus anterior/triceps brachii muscles
C-C VL/BF: Co-contraction between vastus lateralis/biceps femoris muscles
C-C TA/GM: Co-contraction between tibialis anterior/gastrocnemius medialis muscles
DA: Deltoideus anterior muscle
EMG: Electromyography
FW: Free walking
*g*: Gravitational acceleration
GM: Gastrocnemius medialis muscle
KE: Kinetic energy
NW: Nordic walking
PE: Potential energy
*R*: Amount of energy that can be recovered by the pendular exchange between the kinetic and potential energies of the BCoM
*R*(*t*): Instantaneous recovery of mechanical energy by the exchange between the kinetic and potential energies of the BCoM
TA: Tibialis anterior muscle
TB: Triceps brachii muscle
TIDiER: Template for Intervention Description and Replication
VL: Vastus lateralis muscle
*W*_ext_: External work, work done to maintain the movement of the BCoM, it is the sum of the increments in (PE + KE) over a finite number of steps, normalized over a meter of distance traveled
*W*_int_: Internal work, work required to accelerate the body segments relative to BCoM
*W*_int_arms_: Internal work for arms, positive work required to accelerate the upper limbs relative to BCoM
*W*_int_legs_: Internal work for legs positive work required to accelerate the lower limbs relative to BCoM
*W*_int_trunk_: Internal work for trunk, positive work required to accelerate the trunk relative to BCoM
*W*_tot_: total positive work to maintain the locomotion
