# Supplementary material for: Nordic walking training in elderly, a randomized clinical trial. Part II: Biomechanical and metabolic adaptations
Source: Sports Med Open. 2020 Jan 13;6:3. doi: 10.1186/s40798-019-0228-6 (PMC6957599; doi:10.1186/s40798-019-0228-6)
Supplement: Supplementary file 1 — Additional file 1. Template for Intervention Description and Replication (TIDieR). [file 40798_2019_228_MOESM1_ESM.docx]

**Nordic walking training in elderly, a randomized clinical trial. Part II: Biomechanical and metabolic adaptations**

**Sports Medicine Open**

**Natalia Andrea Gomeñuka^1,2^ , Henrique Bianchi Oliveira^1^, Edson Soares da Silva^1^, Elren Passos Monteiro^1,3^, Rodrigo Gomes da Rosa^1^, Alberito Rodrigo Carvalho^1,4^, Rochelle Rocha Costa^1^, Martín Cruz Rodríguez Paz^5^, Barbara Pellegrini^6^, Leonardo Alexandre Peyré-Tartaruga^1^**

***^1^ Exercise Research Laboratory, Universidade Federal do Rio Grande do Sul, Porto Alegre, Brazil.***

***^2^ Departamento de Investigación de la Facultad de Ciencias de la Salud, (UCAMI) Universidad Católica de las Misiones, Posadas, Argentina.***

***^3^ Post-Graduate Program in Health Sciences, Federal University of Health Sciences, Porto Alegre, Brazil.***

***^4^ Physical Therapy College, Universidade Estadual do Oeste do Paraná, Cascavel, Brazil.***

***^5^ Polytechnic Institute San Arnoldo Janssen, Posadas, Argentina.***

***^6^ CeRiSM (Research Centre of Mountain Sport and Health), University of Verona, Rovereto, Italy.***

**e-mail address of the corresponding author: leonardo.tartaruga@ufrgs.br**

**Supplementary Material 01 – Template for Intervention Description and Replication (TIDieR) - Items**

Nordic walking and free walking training: An intervention description including all TIDieR Items (Item 1)

**Rationale (Item 2)**

The Nordic walking is a technique advocate for the development of physical fitness and quality of life due to additional benefits in comparison to free walking. The biomechanical and physiological alterations in walking using poles gives support to our hypothesis that older untrained people training walking using poles will improve the functional mobility, postural balance and quality of life at higher degree than training without poles.

**Materials (Item 3)**

Usual Nordic walking poles were used.

Professionals of physical education taught classes three times per week, at Mondays, Wednesdays, and Fridays, one group training Nordic walking and other free walking.

**Procedures (Item 4)**

Patients in the Nordic walking group were, in addition to usual care, training with poles and in the free walking group without poles.

**The program**

**Framework 01: methodology and didactics for the technique learning of nordic and free walking:**

| **Session** | **Objective** | **Nordic walking** | **Free walking** |
| --- | --- | --- | --- |
| S1 | Posture, strengthening of abdomen and balance (winch) | Posture + Dragging the sticks | Posture |
| S2 | Correction of gait patterns: position of feet, knees and ankles flexion/extension (Squeeze the lemon / kneading grapes) | S1 + Correction of gait patterns: position of feet, knees and ankles flexion/extension (Squeeze the lemon / kneading grapes) | S1 + Correction of gait patterns: position of feet, knees and ankles flexion/extension (Squeeze the lemon / kneading grapes) |
| S3 | Dissociation of pelvic and scapular girdles (Gingado carioca, samba step) | S1 + S 2 + trunk rotation and arm swinging | S1+ S2 + trunk rotation and arm swinging |
| S4 | Coordenation of arms and legs  (hiking in the forest) | S1+S2+S3+ amplitude and arms and legs swinging, with alterning limbs + Pressure of sticks on the ground (load) | S1+S2+S3+  amplitude and arms and legs swinging, with alterning limbs |
| S5 | Range and motion and gait speed (Ayrton Senna) | S1+S2+S3+ S4+ ↑ stride length + Open and closing hands on sticks | S1+S2+S3+S4+ ↑ stride length |
| S6 | Complete technique of walking Técnica completa da caminhada  (fashion week parade) | Technique of Nordic walking | Technique of free walking |

**Note:** S1, S2, S3, S4, S5, S6 = Sessions of familiarization; ↑ = increase; Words in () = the linguage of connection used. From MONTEIRO, E. P. 2014. 226 pages. Dissertation of Master Science in Human Movement Sciences – Post-Graduate Program in Human Movement Sciences, Department of Physical Education, Universidade Federal do Rio Grande do Sul, Porto Alegre, 2014.

**Framework 2. Stretching exercises.**

| **STRETCHING** | | **Why aerobic activities like walking?**  • Reduces the risks of cardiovascular diseases. • Improves strength, endurance, coordination and flexibility. • Improves mood.  **Why stretch?**  **•** Relaxes muscles by reducing fatigue.  • Decreases joint pressure.  • Improves body posture.  • Helps increase muscle strength.  **Guidance for stretching and walking exercises**  **•** A minimum duration of 20 seconds in each stretching exercise.  • Always keep the spine straight.  • Keep your eyes on the horizon.  • Stretching every day allows for a better result.  • Perform the exercises in the "ON" state of the medicine, for greater mobility.  • Warm joints with joint movements  • Walk 2 to 3 times a week for 20 to 30 minutes.  • After the walk stretch lower limbs.  • Monitor fatigue after exercise. You may feel tired, but not exhausted.  • Remember to alternate arms and legs to walk.  • Hydrate before, during and after walking.  • During the walk, the first contact of the foot with the ground should be that of the heel.  • The first step of the walk is always the longest, to avoid the episode of freezing. |
| --- | --- | --- |
| 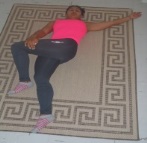 | Lying on the floor or bed you should extend one knee, flex the other knee and cross to the other side. Feel the gluteus stretch. |  |
| 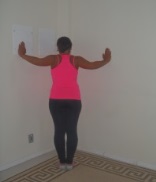 | You should locate a corner of a wall and keep your arms shoulder-length and protrude forward. Feel to stretch the chest |  |
| 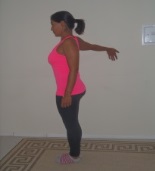 | Standing put your palm up, press against the wall. Feel your arm stretch. The hand can not be above the shoulder and they can not hurt. |  |
| 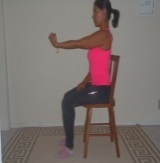 | Sitting stretched out the elbow and flexed the fist. Feel the lengthening of the forearm. |  |
| 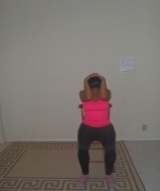 | Sitting, flex the neck holding the head with both hands, elbows close. Feel the length of your neck. |  |
| 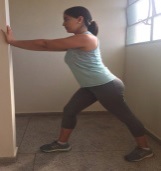 | Hands leaning against the wall. Straight legs, one knee flexed forward and one extended knee. Feel the length of the back of the leg. |  |
| 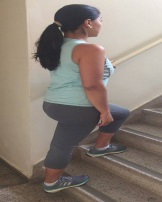 | Standing on a ladder or step, place one foot forward with knee flexed and the other foot supported lowering the heel, with the leg extended. Stretch the calf. |  |
| 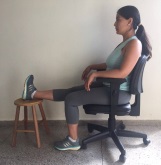 | Sitting with the spine straight, one leg should be resting on the floor, and the other leg lying on a bench, feeling stretching the back of the thigh. |  |
| 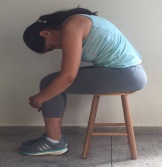 | Sitting on a bench, flex your torso forward, so that your hands embrace the legs, feeling stretch the lumbar and thoracic. |  |
| 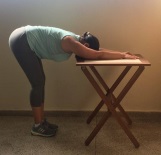 | Standing, with your feet together and aligned, bend your torso forward, hands resting on the table, the spine should be straight, the head between the arms, stretching the back of the thigh, trunk and arms. |  |

Note: From MONTEIRO, E. P. 2014. 226 pages. Dissertation of Master Science in Human Movement Sciences – Post-Graduate Program in Human Movement Sciences, Department of Physical Education, Universidade Federal do Rio Grande do Sul, Porto Alegre, 2014.

**Figure 01:** Example of Nordic walking class on athletics track and gymnasium of School of Physical Education, Physical Therapy and Dance of Universidade Federal do Rio Grande do Sul, Brazil.


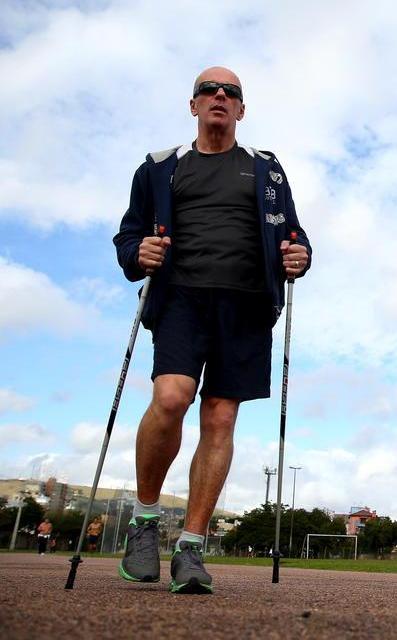

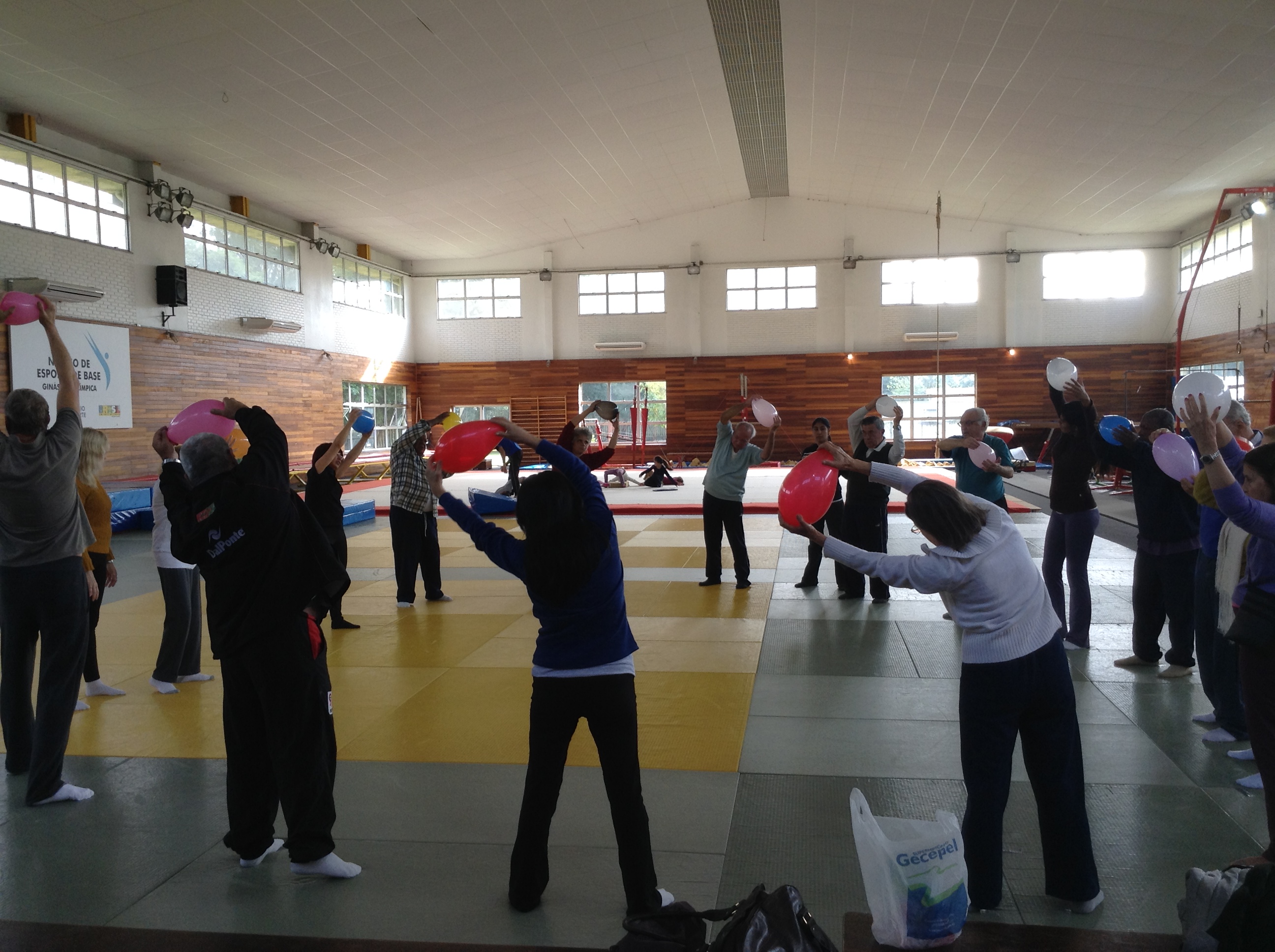


**Providers (Item 5)**

The intervention is primarily provided by the primary investigator, a professional of Physical Education with 5 years of clinical experience, trained in providing the intervention throughout the development phase and in pilot testing of the intervention. Alternates designated to take over in case the primary investigator is unable to complete one or more intervention sessions will be professionals of Physical Education and Physiotherapists trained and approved by the primary investigator. Training was focus on uniform correction of exercise form, progression and regression of exercises and standard face-to- face adherence reminders.

**How (Item 6)**

The intervention (being instructions in the exercise program) is delivered face-to-face individually to each patient.

**Where (Item 7)**

The face-to-face instructions are provided at the study site, in an athletics track. Exercises were performed also at athletics track and gymnasium.

**When and how much (Item 8):**

The training period had eight weeks in duration, with three weekly sessions (24 sessions in total). The volume (session time in minutes) and intensity (percentage of heart rate at the second ventilatory threshold reached by the participants during the sessions) were equal for both groups. HR was controlled by a cardiac monitor (POLAR, S610 model, Finland) during training sessions, with the groups only differing by the use (or not) of the poles during the walks. Training periodization in the different sessions is described as follows: in the warming-up, all sessions started with 5 minutes of warming-up; then followed the main part, with the time referring to the aims of volume and intensity of the session (Fig 3, Training Periodization in the main document); finally the back to calm was held, all sessions ended with five minutes of back to calm and posteriorly stretching was performed.

**Tailoring (Item 9)**

For all intervention Phases the intensity and volume was individualized, respecting the principles of physical training (individuality, adaptation, progression, specificity, continuity).

**Modifications (Item 10)**

In case of modifications to the intervention during the study period, these will be reported in the primary trial report.

**Adherence and fidelity (Item 11)**

Lack of adherence to an exercise intervention is a major problem when aiming at investigating the effect of an intervention. All participants had a minimal adherence of 90%, thus demonstrating the effectiveness of the training program.

References:

Hoffmann TC, Glasziou PP, Boutron I, Milne R, Perera R, Moher D, et al. Better reporting of interventions: template for intervention description and replication (TIDieR) checklist and guide. BMJ. 2014;348:g1687.

Arcila DMC, Monteiro EP, Gomeñuka NA, Peyré-Tartaruga LA. Methodology and pedagogical didactics applied to the education of nordic walking and free walking for people with Parkinson’s disease I. Cadernos de Formação RBCE, 8, 2, 2017. In: revista.cbce.org.br/index.php/cadernos/article/view/2265

This is an Additional File for the article titled:

Nordic Walking training in elderly, a randomized clinical trial: Part II: Biomechanical and metabolic adaptations
